# Le syndrome de Mayer-Rokitansky-Küster-Hauser comme cause d'aménorrhée primaire: à propos d'un cas

**DOI:** 10.11604/pamj.2021.40.260.29181

**Published:** 2021-12-23

**Authors:** Mohamed Abdellaoui, Jamal El Fenni, Meryem Edderai

**Affiliations:** 1 Service de Radiologie, Hôpital d'Instruction Militaire Rabat, Rabat, Maroc,; 2 Service d'Imagerie Médicale, Hôpital Militaire Mohammed V, Rabat, Maroc

**Keywords:** Aménorrhée primaire, aplasie utéro-vaginale, IRM, cas clinique, Primary amenorrhea, uterine vaginal aplasia, MRI, case report

## Abstract

Le syndrome de Mayer-Rokitansky-Küster-Hauser (MRKH) est une cause rare d´aménorrhée primaire. Il est défini par une aplasie congénitale de l´utérus et des deux tiers supérieurs du vagin chez des femmes présentant un développement normal des caractères sexuels secondaires. Le diagnostic est basé essentiellement sur imagerie par résonance magnétique (l´IRM). Nous rapportons le cas d'une fille de 17 ans qui a consulté pour une aménorrhée primaire, avec des caractères sexuels secondaires présents et bien développés. Le bilan biologique a révélé une fonction ovarienne normale ainsi que l´axe gonadotrope. L´échographie pelvienne et l´imagerie par résonance magnétique ont mis en évidence une agénésie complète de l´utérus, des deux tiers supérieurs du vagin et du rein gauche permettant de confirmer le diagnostic du syndrome de Mayer-Rokitansky-Küster-Hauser (MRKH) type II. L´intérêt de ce cas clinique est d'évoquer le diagnostic de MRKH, Devant toute aménorrhée primaire chez une jeune femme présentant des caractères sexuels bien développés, et aussi de chercher les signes spécifiques sur l'imagerie notamment l'IRM.

## Abstract (en)

Mayer-Rokitansky -Küster -Hauser syndrome (MRKH) is a rare cause of primary amenorrhea. It is defined as congenital aplasia of the uterus and of the upper two thirds of the vagina in women with normal development of secondary sexual characteristics. Diagnosis is essentially based on magnetic resonance imaging (MRI). We here report the case of a 17-year-old girl presenting with primary amenorrhea with well developed secondary sexual characteristics. Laboratory tests revealed normal ovarian function as well as gonadotropic axis. Pelvic ultrasonography and magnetic resonance imaging revealed complete uterine agenesis of the uterus, the upper two-thirds of the vagina and left kidney, allowing to confirm the diagnosis of Mayer-Rokitansky-Küster-Hauser syndrome type II. This study highlights the importance of suspecting MRKH in young patients with well-developed sexual characteristics and any type of primary amenorrhea as well as of performing MRI in order to detect any specific sign of this disorder.

## Introduction

L´aménorrhée primaire correspond à l'absence de règles à l'âge de 15 ans chez des patientes qui ont une croissance normale et des caractères sexuels secondaires. Le syndrome de Mayer-Rokitansky-Küster-Hauser (MRKH) reste une cause rare de l´aménorrhée primaire [1], il est défini par une aplasie congénitale de l´utérus et des deux tiers supérieurs du vagin chez des femmes présentant un développement normal des caractères sexuels secondaires et un caryotype normal (46, XX) [2]. Nous rapportons le cas d'une fille de 17 ans qui a consulté pour une aménorrhée primaire, sans anomalie clinique ni biologique, chez qui l´échographie pelvienne et l´imagerie par résonnance magnétique ont révélé le syndrome de Mayer-Rokitansky-Küster-Hauser.

## Patient et observation

**Information de la patiente:** Il s´agit d´une fille de 17 ans, sans antécédents particuliers, qui a consulté pour une aménorrhée primaire.

**Résultats cliniques:** l´examen clinique a mis en évidence des organes génitaux externes de phénotype féminin, des seins bien développés avec présence d´autres caractères sexuels secondaires. La patiente était vierge d'où la non réalisation du toucher vaginal.

**Figure 1:**
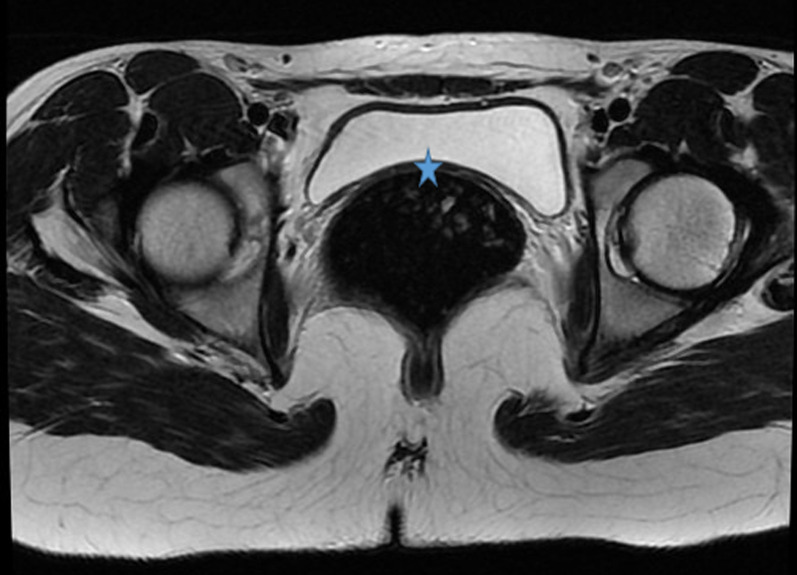
IRM pelvienne; séquence T2 coupe axiale: agénésie utérine (étoile)

**Figure 2:**
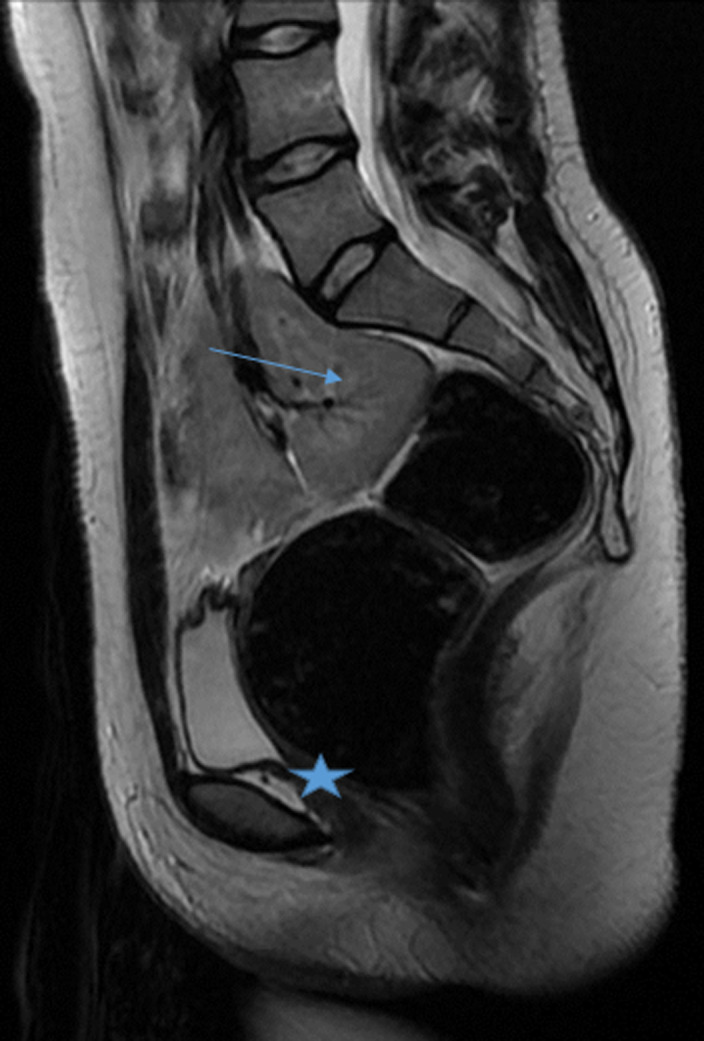
IRM pelvienne: séquence T2 coupe Sagittale; agénésie utérine et 2/3 supérieur du vagin (étoile), associé à un rein ectopique pelvien (flèche)

**Figure 3:**
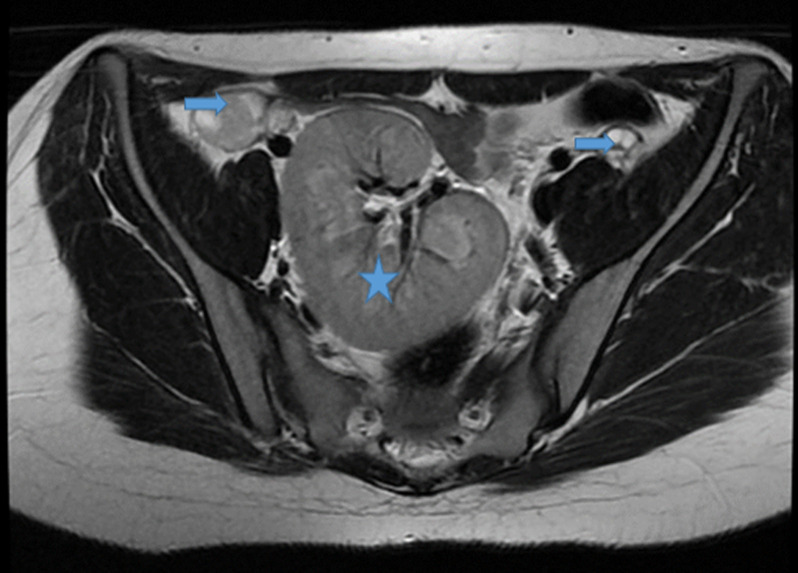
IRM pelvienne: séquence T2 coupe Axiale: morphologie et taille normale des ovaires (flèches), rein droit ectopique pelvien (étoile)

**Figure 4:**
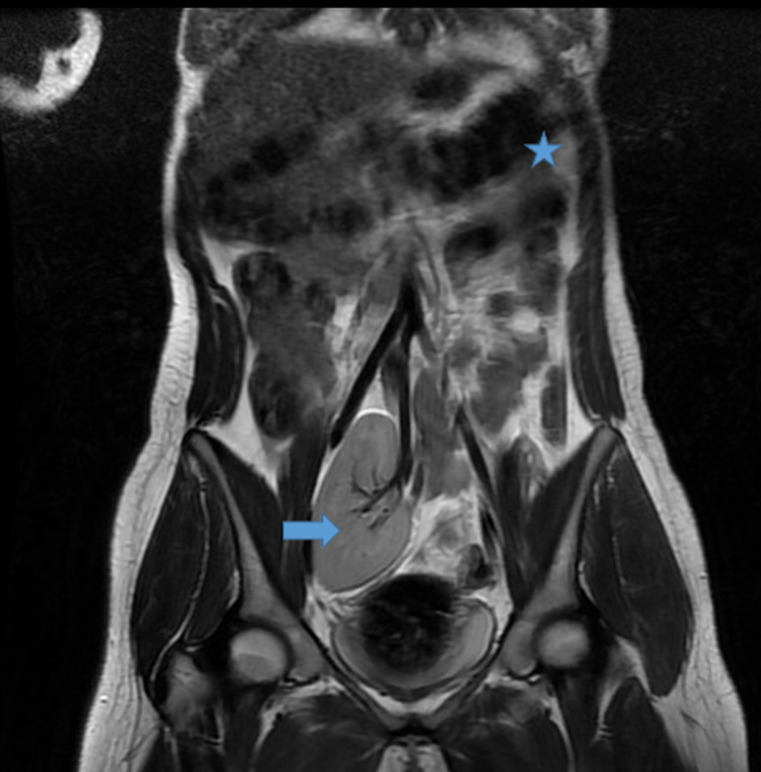
IRM pelvienne: séquence T2 coupe coronale: agénésie rénale gauche (étoile), rein droit ectopique pelvien (flèche)

**Démarche diagnostique:** le bilan hormonal a confirmé une fonction ovarienne normale avec une 17-esradiol à 100 pg/ml, ainsi que l´axe gonadotrope avec une FSH à 6 mUI/ml et LH à 4 mUI/ml et un dosage normal de la testostérone. L´étude génétique a mis en évidence un caryotype constitutionnel à 46 XX. L´échographie et l´IRM pelvienne ont montré l´agénésie complète de l´utérus et des deux tiers supérieurs du vagin (Figure 1, Figure 2), l'aspect et le signal normal des ovaires (Figure 3), l'agénésie du rein gauche (Figure 4) et l'ectopie pelvienne du rein droit (Figure 2, Figure 3, Figure 4), permettant ainsi de poser le diagnostic du syndrome de Mayer-Rokitansky-Küster-Hauser type II.

**Intervention thérapeutique et suivi:** la patiente a bénéficié d'un soutien psychologique, et afin d´assurer une vie sexuelle normale, elle sera programmée, ultérieurement, pour une vaginoplastie sous cœlioscopie.

## Discussion

Le syndrome de Mayer-Rokitansky-Küster-Hauser (MRKH) est défini par une aplasie congénitale de l´utérus et des deux tiers supérieurs du vagin chez des femmes présentant un développement normal des caractères sexuels secondaires et un caryotype normal (46, XX) [2]. Deux formes cliniques sont décrites: MRKH de type I qui correspond à une agénésie utérovaginale isolée et MRKH de type II qui se caractérise par une agénésie incomplète et/ou associée à d´autres malformations congénitales [3]. Pour notre patiente il s´agit d´une MRKH de type II. L´incidence est estimée à une femme sur 4500 [1]. Le signe clinique principal est l´aménorrhée primaire, les caractères sexuels secondaires sont présents et bien développés [4]. Le bilan génétique montre un caryotype sanguin normal (46, XX) sans anomalie chromosomique visible [5]. Le bilan endocrinien (FSH, LH et 17-estradiol plasmatiques) est normal, témoignant de l´intégrité de la fonction ovarienne ainsi que l´axe gonadotrope (FHS, LH) [5]. Chez notre patiente le bilan hormonale et génétique étaient normaux. L´échographie par voie sus-pubienne est un moyen de première intention, elle permet de suspecter le diagnostic en montrant une absence de structure utérine entre la vessie et le rectum. Néanmoins, une structure quadrangulaire rétro vésicale peut être identifiée à tort comme utérus hypoplasique, elle correspond à la lame vestigiale située sous la partie médiane du repli péritonéal transversal. Une malformation rénale associée doit, par ailleurs, être systématiquement recherchée au cours de cette échographie [6].

L´IRM est un examen plus sensible et plus spécifique que l´échographie sus-pubienne. Elle permet un diagnostic précis grâce à la séquence T2 en coupe sagittale et axiale, confirmant L´aplasie utérine et les deux tiers supérieurs du vagin, et l´aspect normal des deux ovaires [7]. L´IRM permet, par ailleurs, la recherche d´autres malformations associées (rénales et osseuses). Devant une patiente présentant une aménorrhée primaire avec des caractères sexuels secondaires bien différenciés le diagnostic différentiel se posera avec tout d´abord L´atrésie vaginale ou une cloison vaginale transverse, le diagnostic sera confirmé par un examen clinique attentif et la présence d´un utérus à l´imagerie [8,9]. Ensuite Le syndrome de mutation du gène WNT4, Le phénotype est très proche du MRKH avec une aménorrhée primaire, une aplasie utérovaginale avec éventuellement une malformation rénale. Ces anomalies sont toutefois associées à des signes d´hyperandrogénie (acné et hirsutisme), corrélés par des dosages plasmatiques montrant une testostéronémie élevée [10]. Enfin Le syndrome d´insensibilité aux androgènes, C´est un pseudohermaphrodisme masculin. Le phénotype est féminin avec présence de testicules en position abdominale ou inguinale et un taux de testostérone élevé équivalent à celui du sujet masculin. Le traitement consiste en la reconstitution d'un néovagin, permettant à la patiente d'avoir une vie sexuelle normale. Un soutien psychologique s'avère primordial pour les patientes atteintes d'un syndrome de MRKH [10].

## Conclusion

Devant une aménorrhée primaire chez une jeune femme présentant des caractères sexuels physiques bien développés, le syndrome de Mayer-Rokitansky-Küster-Hauser (MRKH) doit être évoqué. Le diagnostic est basé essentiellement sur l´imagerie notamment l´échographie et l´IRM pelvienne. Cette dernière est l´examen de choix permettant, grâce à la séquence T2, de confirmer l´aplasie utéro-vaginale, l´intégrité des ovaires et aussi la recherche des malformations associés notamment rénale. Néanmoins La confusion possible avec d´autres syndromes incluant une anomalie utérovaginale nécessite une connaissance des différents diagnostics différentiels.
